# Characterization of Chromosomal Instability in Murine Colitis-Associated Colorectal Cancer

**DOI:** 10.1371/journal.pone.0022114

**Published:** 2011-07-22

**Authors:** Marco Gerling, Rainer Glauben, Jens K. Habermann, Anja A. Kühl, Christoph Loddenkemper, Hans-Anton Lehr, Martin Zeitz, Britta Siegmund

**Affiliations:** 1 Medical Clinic I, Charité – Universitätsmedizin Berlin, Campus Benjamin Franklin, Berlin, Germany; 2 Laboratory for Surgical Research, Department of Surgery, University of Lübeck, Lübeck, Germany; 3 Institute of Pathology / RCIS, Charité – Universitätsmedizin Berlin, Campus Benjamin Franklin, Berlin, Germany; 4 Centre Hospitalier Universitaire Vaudois (CHUV), Institut Universitaire de Pathologie, Lausanne, Switzerland; Institut Jacques Monod, France

## Abstract

**Background:**

Patients suffering from ulcerative colitis (UC) bear an increased risk for colorectal cancer. Due to the sparsity of colitis-associated cancer (CAC) and the long duration between UC initiation and overt carcinoma, elucidating mechanisms of inflammation-associated carcinogenesis in the gut is particularly challenging. Adequate murine models are thus highly desirable. For human CACs a high frequency of chromosomal instability (CIN) reflected by aneuploidy could be shown, exceeding that of sporadic carcinomas. The aim of this study was to analyze mouse models of CAC with regard to CIN. Additionally, protein expression of p53, beta-catenin and Ki67 was measured to further characterize murine tumor development in comparison to UC-associated carcinogenesis in men.

**Methods:**

The AOM/DSS model (n = 23) and IL-10^−/−^ mice (n = 8) were applied to monitor malignancy development via endoscopy and to analyze premalignant and malignant stages of CACs. CIN was assessed using DNA-image cytometry. Protein expression of p53, beta-catenin and Ki67 was evaluated by immunohistochemistry. The degree of inflammation was analyzed by histology and paralleled to local interferon-γ release.

**Results:**

CIN was detected in 81.25% of all murine CACs induced by AOM/DSS, while all carcinomas that arose in IL-10^−/−^ mice were chromosomally stable. Beta-catenin expression was strongly membranous in IL-10^−/−^ mice, while 87.50% of AOM/DSS-induced tumors showed cytoplasmatic and/or nuclear translocation of beta-catenin. p53 expression was high in both models and Ki67 staining revealed higher proliferation of IL-10^−/−^-induced CACs.

**Conclusions:**

AOM/DSS-colitis, but not IL-10^−/−^ mice, could provide a powerful murine model to mechanistically investigate CIN in colitis-associated carcinogenesis.

## Introduction

Patients suffering from ulcerative colitis (UC) face an increased lifetime risk of developing colorectal cancer (CRC) [1]. Such inflammation-associated malignancies of the colorectum show distinct differences to sporadic carcinomas: they develop in younger patients, more often in males, and synchronous carcinomas are more frequently found [2]. On the genomic level, it has been hypothesized that chronic inflammation leads to increased chromosomal instability (CIN) by reactive oxygen and nitrogen species (RONS), hypermethylation of pericentromeric DNA regions, telomere attrition, and other less well defined mechanisms [3], [4], [5]. CIN is observed in chronic inflammatory conditions such as Barrett's esophagus, chronic hepatitis, and UC to a high extent [6], [7], [8]. In UC, aneuploidy as the measurable sequela of CIN can be applied as a predictive marker for malignant transformation and is detectable up to a decade prior to diagnosis of carcinoma [8], [9]. Recently, it could be shown that CIN characterizes colitis-associated carcinomas (CACs) with a frequency reaching 100% in a set of 31 CACs analyzed, while contrarily only 75% of sporadic CRCs were found aneuploid [10], [11]. Taken together, mounting evidence suggests a causal relationship between inflammation and CIN, with presence of CIN being a predictive marker for both, malignancy development and inferior prognosis once malignant transformation has occurred.

Elucidating causes and effects of CIN on a mechanistic level could therefore substantially aid the development of strategies to prevent and treat cancer with novel, targeted approaches. Thus, suitable animal models are highly desirable to accelerate research progress. Preferably, such models should show characteristics similar to their human counterparts, which in case of colitis-associated carcinogenesis indispensably comprise aneuploidy.

In addition to CIN, previous studies have demonstrated further differences between sporadic and colitis-associated carcinogenesis with regard to canonical pathways of malignant transformation:

It has long been known that p53-point mutations occur early in UC-associated neoplastic progression and correlate directly with aneuploidy [12], [13], [14], [15].

In CRC and other tumors, activation of Wnt-signaling promotes cell survival and inhibits cell death. Subsequent to activation of the Wnt-signaling pathway, accumulation and translocation of beta-catenin from the cell membrane to the cytoplasm and nucleus can be observed, resulting in activation of a variety of target genes [16]. Only limited data exist on beta-catenin-expression in CAC. One study focusing on genetic alterations adjacent to the beta-catenin locus on chromosome 3p22-p21.3 could not find a difference between the frequency of loss of heterozygosity among UC-associated and sporadic carcinomas [17]. Contrarily, a recent study on Wnt-signaling activation in CAC concluded that the pathway is activated in an early phase of malignant transformation in colitis, and found nuclear beta-catenin staining helpful in detecting neoplasia in CAC [18].

A diversity of animal models of UC is commonly used. In one canonical model colitis is induced with dextran sulphate sodium (DSS) [19]. Interestingly, long term DSS administration alone can cause malignant transformation in rodents [20], [21], while this effect is aggravated by additional application of azoxymethane (AOM), a mutagenic agent that by itself causes the development of colorectal tumors in mice [22]. Tumors induced with AOM alone do not show CIN [23]. A dose-dependent promoting effect of DSS for AOM-induced tumors has been reported, while CIN has not been studied in the AOM/DSS-model [24].

A complementary murine model of UC is the interleukin 10^−/−^ (IL-10^−/−^)-mouse [25]. In this model, inflammatory changes commence in the distal colon at about three weeks of age and progress proximally without additional administration of external pathogens [26]. Colonic lesions are characterized by inflammatory infiltrates in the mucosa and submucosa as well as crypt abscesses [26]. It has been described that at six months of age, 60% of IL-10^−/−^-mice develop adenocarcinomas [26]. We have previously shown that lumen filling tumors arise in IL-10^−/−^ mice, which closely resemble human adenocarcinomas histologically [27]. Furthermore, it has been reported that neoplastic transformation in these mice can be aggravated by administration of celecoxib [25], [27].

The aim of this project was to characterize murine inflammation-associated CRCs with regard to CIN and other known characteristics of their human counterpart. The extent of CIN in premalignant and malignant stages of two mouse models for colitis-mediated carcinogenesis was assessed. Furthermore, the protein expression of p53 and beta-catenin was evaluated in murine tumors, as well as in an exemplary set of human CACs. Ki67 staining served to determine the growth fraction of the tumors. Finally, inflammatory activity was confirmed via endoscopy, histology, as well as a local increase of the pro-inflammatory cytokine interferon-γ (IFNγ)

## Materials and Methods

### Ethical Considerations

Animal protocols were approved by the regional animal study committee of Berlin (LAGeSo, approval ID G0297/03) for both models used in this study.

### Mice

All animals were purchased from Harlan Winkelmann (Borchen, Germany). Altogether, 23 mice treated with AOM and DSS as described below were used for this study. Complementarily, eight IL-10^−/−^ mice were investigated.

### AOM/DSS-induced tumor development

DSS and AOM were administered to C57BL/6J mice at six weeks of age as described previously [27]. Briefly, mice received a single intraperitoneal injection of the mutagenic agent AOM (12.5 mg/kg body weight). Starting at day five after the AOM application, 3.5% DSS was dissolved in the drinking water for three cycles of five days each with intermittent 14-day intervals of regular drinking water, thereby inducing a chronic DSS colitis. Endoscopic surveillance was performed as described below on a weekly basis and mice were sacrificed on day 50, at that point exhibiting either adenocarcinomas or colitis without overt neoplasms (see results, **table 1**).

**Table 1:** Ploidy assessment according to Auer's classification, p53-, beta-catenin-, and Ki67-immunohistochemistry for murine tissue analyzed.

No.	Auer	Aneuploid	p53	b-catenin	Ki67 / %
*AOM-CA1*	4	1	2	4	30
*AOM-CA2*	4	1	1	4	20
*AOM-CA3*	4	1	1	4	25
*AOM-CA4*	4	1	2	1	10
*AOM-CA5*	4	1	1	4	30
*AOM-CA6*	4	1	1	4	25
*AOM-CA7*	4	1	1	1	10
*AOM-CA8*	4	1	1	2	10
*AOM-CA9*	4	1	2	3	20
*AOM-CA10*	4	1	2	2	35
*AOM-CA11*	4	1	3	2	10
*AOM-CA12*	3	0	3	3	15
*AOM-CA13*	3	0	1	4	20
*AOM-CA14*	4	1	1	5	5
*AOM-CA15*	3	0	2	4	40
*AOM-CA16*	4	1	3	4	40
*IL10-CA1*	3	0	3	1	40
*IL10-CA2*	3	0	3	1	40
*IL10-CA3*	3	0	3	1	35
*IL10-CA4*	3	0	3	1	40
*AOM-CNTRL1*	3	0	1	2	20
*AOM-CNTRL2*	1	0	0	1	5
*AOM-CNTRL3*	1	0	1	1	5
*AOM-CNTRL4*	3	0	1	1	10
*AOM-CNTRL5*	3	0	2	1	15
*AOM-CNTRL6*	1	0	1	2	5
*AOM-CNTRL7*	3	0	2	2	15
*IL10-CNTRL1*	3	0	3	1	35
*IL10-CNTRL2*	3	0	2	1	35
*IL10-CNTRL3*	3	0	2	1	30
*IL10-CNTRL4*	3	0	2	1	25

AOM-CA_n_: carcinomas of AOM/DSS-colitis. IL10CA_n_: carcinomas of IL10^−/−^-mice. CNTRL_n_: premalignant tissue.

DNA-ploidy was assessed according to Auer (please refer to the text). p53 was assessed semiquantitatively, 0: no expression, 1 1–20% positive mucosa cells, 2: 21–50%, 3>50%

Beta-catenin: 1: membranous, 2: membranous-cytoplasmatic, 3: cytoplasmatic, 4: cytoplasmatic-nulcear, to 5, nuclear.

**Figure 1:**
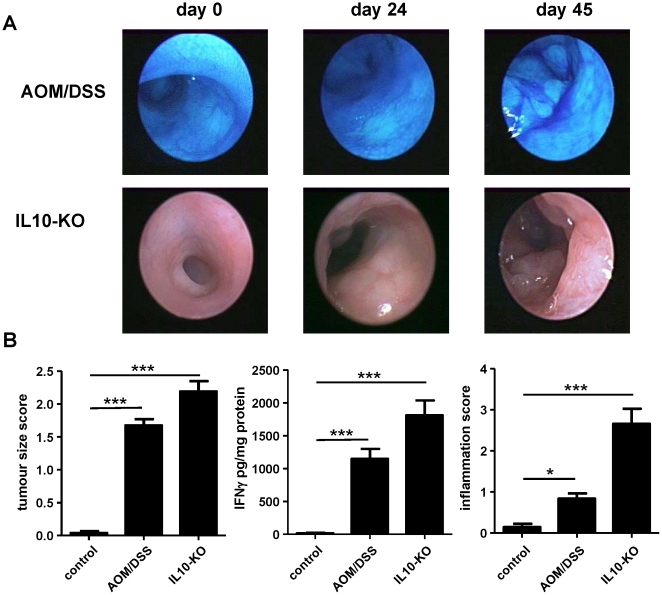
Murine models of inflammation-driven carcinogenesis. (A) Endoscopic images of inflamed mucosa and tumor of AOM/DSS-colitis as well as the IL10^−/−^ model. Tumorigenesis was assessed by high resolution endoscopy *in vivo*. Methylene blue was used to enhance dysplasia detection as shown exemplarily for AOM/DSS colitis (B) Tumor size as rated by the tumor size score described in the methods section, IFNγ -levels, and inflammation score as assessed in H.E. staining for untreated controls, AOM/DSS-mice, and IL10^−/−^ mice. Bars represent means, whishkers represent standard error of the mean (SEM). Colonic mucosa samples of 10 healthy untreated C57BL/6J-mice were applied to assess histologic parameters and IFNγ -expression.

In total, ten healthy, untreated C57BL/6J-mice were used as normal controls (**figure 1**).

### IL-10^−/−^-mice

At twelve weeks of age, the cyclooxygenase 2 inhibitor celecoxib (500 mg/mouse/day) was administered orally to IL-10^−/−^ mice (C57BL/6J –background, n = 8) for five days as described previously [25]. Mice were observed for an additional four weeks and underwent weekly lower endoscopy, before being sacrificed with or without signs of malignant growth (**table 1**).

### Endoscopic surveillance and post-mortem examination

Tumor development was monitored macroscopically using a high resolution mouse endoscope system (Karl Storz GmbH, Tuttlingen, Germany) as described previously [28]. Endoscopies were performed weekly starting one week after AOM-treatment and celecoxib-treatment, respectively. Based on observed tumor development, days 50 and 28 after beginning of each treatment were chosen as endpoint for the AOM/DSS and IL-10^−/−^ group, respectively. Endoscopic procedures surveyed on a color monitor were recorded digitally (DSR-20MD, Sony, Cologne, Germany).

In addition to preparation of colonic tissue as described below, post-mortem autopsy was performed macroscopically. Particularly, to screen for distant metastases, fresh lung and liver tissue was cut into 5 mm sections, which were investigated for macroscopical signs of neoplasms.

### DNA-image cytometry

Nuclear DNA ploidy assessments were performed by means of DNA-image cytometry using Feulgen-stained histological sections of 8 µm thickness. Staining procedures, cell selection criteria, and internal standardization were based on methods described previously [29]. An average number of 110 enterocyte nuclei (range 100 to 120, SD  = 3.4) were measured per specimen after interactive selection using a digital imaging system (Ahrens ACAS, Hamburg, Germany). All DNA values were expressed in relation to internal staining controls (lymphocytes), which were given the value 2c. DNA profiles were classified according to Auer [29]. Histograms characterized by a single peak in the diploid or near-diploid region (1.5 c–2.5 c) were classified as type I. Type II histograms showed a single peak in the tetraploid region (3.5 c–4.5 c) or peaks in both the diploid and tetraploid regions (>90% of the total cell population). The number of cells with DNA values between the diploid and tetraploid region and those exceeding the tetraploid region (>4.5 c) was <10%. Type III histograms represented highly proliferating near-diploid cell populations and were comprised of DNA values ranging between the diploid and the tetraploid region. Only a small number of cells (<5%) showed more than 4.5 c. The DNA histograms of types I, II and III thus characterize euploid cell populations. Type IV histograms showed increased (>5%) and/or distinctly scattered DNA values exceeding the tetraploid region (>4.5 c). These histograms were suggested to reflect aneuploid populations of enterocyte nuclei.

### Colon organ culture and cytokine measurements

Murine colons were cut open longitudinally and washed in PBS. Strips of 1 cm^2^ were placed in 48 flat-bottom well culture plates containing 0.5 ml of serum-free RPMI 1640 with penicillin (100 U/ml) and streptomycin (100 µg/ml), and were incubated at 37°C for 24 h. Culture supernatants were harvested, assayed for IFNγ, and total protein content was quantified using the Bio-Rad protein assay (Bio-Rad Laboratories, Munich, Germany). Murine IFNγ was determined by specific ELISA according to the manufacturer's protocols (BD Biosciences, Heidelberg, Germany) with a quantification range from 20 pg/ml to 10 ng/ml.

### Histopathology and Immunohistochemistry

All tumors and mucosa specimens were subjected to *Hematoxylin* & *Eosin* (H&E) staining for histopathology assessment by an experienced pathologist (H.-A. L). From AOM/DSS-treated mice or IL-10^−/−^ mice, complete colons were fixed. Paraffin sections were stained with H&E. Histological signs of inflammation were evaluated as a combined score of inflammatory cell infiltration (0–3) and tissue damage (0–3), resulting in a score ranging from 0 to 6 as described previously [30]. Tumor size was scored as follows: 0, no adenoma; 1, focal; 2, expanded and/or confluent; 3, expanded and raised; and 4, lumen filling. Antibodies for p53, beta-catenin, and Ki67 were purchased from DAKO (Hamburg, Germany; anti-Ki67 antibody; clone TEC3; rat IgG2a), New England Biolabs (Frankfurt, Germany; anti-beta-catenin antibody; clone 6B3, rabbit IgG), and Cell Signaling (Danvers, MA, USA; anti-p53 antibody, Ser15; polyclonal rabbit). Staining procedures were performed according to the manufacturers' protocols. At least ten high power fields were used to semiquantitatively assess protein expression. For immunohistochemistry scoring please refer to **table 1**.

### Statistical Analyzes

Statistical data analyzes were performed using Microsoft Excel 2003, DigDB v7.1.3.3 (Sunnyvale, CA, USA), and XLStat Pro v7.5 (Addinsoft, New York, NY, USA). Data are expressed in means (using standard error of means, SEM). For inductive inference, nonparameteric rank-sum tests were used to compare location parameters of data distributions. Two independent samples, e.g. of different ploidy status, were compared using Wilcoxon's test. Corresponding frequencies were analyzed by means of Fisher's exact test. The type 1 error rate was set to 5%.

## Results

### Administration of AOM/DSS and IL-10 deficiency result in colitis-mediated carcinomas

In total, 16 out of 23 mice treated with AOM and DSS developed colorectal neoplasms after an average age of seven weeks post AOM administration. Tumor development could be followed endoscopically and tumors closely resembled human adenocarcinomas histologically. In mice that developed neoplasms (n = 16), tumor number ranged from two to 19 per animal with a median of nine tumors per mouse (**figure 1a**). From each individual, one representative tumor was chosen for further analyses on the basis of tumor size. However, in only one out of 16 cases, invasion through the colonic wall was observed, while all other carcinomas were characterized by obstructing intraluminar growth without infiltration beyond the lamina propria mucosae. Neoplasms were seen exclusively in the colon.

In IL-10^−/−^ mice, at an average age of 15 weeks upcoming dysplasias progressed to infiltrating adenocarcinomas in a total of four animals (out of eight animals observed as described in “methods”). Tumor number ranged from seven to 24 tumors per animal, with a median of 16 neoplasms per mouse. One tumor of each animal was chosen for downstream analyses as described above. Neoplastic transformation was constrained to the colorectum.

Macroscopic distant metastases were seen neither after AOM/DSS-treatment nor in IL-10^−/−^ mice.

### Chronic intestinal inflammation in both models as prerequisite for tumorigenesis

Chronic inflammation at the site of tumorigenesis was characterized by a significant increase of the histological inflammation score in parallel to elevated IFNγ levels in the colon culture supernatants of both models when compared to healthy controls (f**igure 1b**).

### IL-10^−/−^ colitis results in chromosomally stable tumors

**Figure 2:**
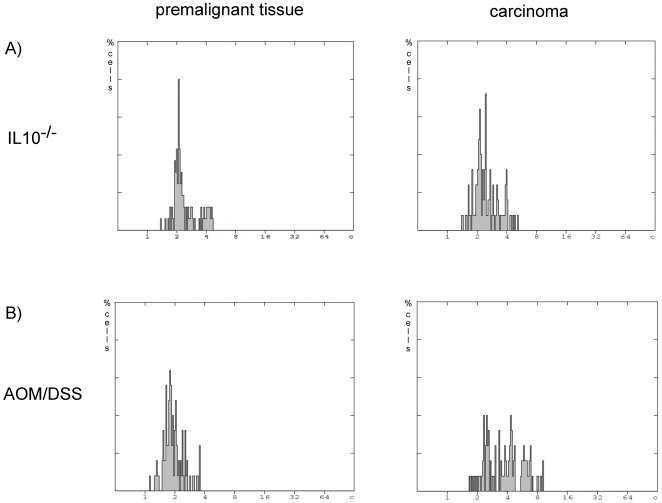
DNA-histograms. (A) Representative DNA histograms on logarithmic scale of premalignant and malignant tissue of IL-10^−/−^ mice. Both histograms depict diploid proliferative patterns. (B) Mice treated with AOM/DSS showed diploid-proliferative patterns in premalignant stages. In the here presented example of CAC, 23% of all cells measured exceeded the threshold of 4.5 c, representing an aneuploid cell population.

A total of four IL-10^−/−^ mice developed invasive colorectal carcinomas devoid of CIN. **Figure 2a** shows a DNA-histogram of inflamed mucosa and an example of an invasive carcinoma in IL-10^−/−^ mice, both depicting a diploid-proliferative pattern. Histograms indicate a proliferating tumor without gross instability of the genome. In total, all four carcinomas of IL-10^−/−^ mice presented diploid patterns. Likewise, all non-neoplastic mucosa samples showed diploid histograms. All non-neoplastic mucosa samples that were applied for DNA-cytometry and IHC presented with signs of chronic inflammation (average inflammation score of 2.1), while absence of neoplasia was ensured histologically (tumor score  = 0).

### AOM induced tumors that arise in mice treated with DSS show signs of gross CIN

Non-malignant mucosa after administration of AOM and DSS revealed a diploid pattern in DNA-cytometry (n = 7/7). Chronic inflammation was present in all non-neoplastic biopsies with an average inflammation score of 0.5, tumor score  = 0.

Overt colorectal neoplasms on the basis of AOM/DSS-colitis presented aneuploidy with a significant amount of cells exceeding 4 c (f**igure 2b**). In total, 13 out of 16 carcinomas arising in AOM/DSS-colitis showed aneuploidy reflecting CIN. The frequency of aneuploidy differed significantly between AOM/DSS-induced tumors and IL-10^−/−^-induced tumors (p = 0.007).

### Protein expression of CRC-associated gene products differs between both tumor types

In all IL-10^−/−^ carcinomas (n = 4), membranous beta-catenin localization was found, while this was the case for two out of 16 AOM/DSS-induced tumors. In nine out of 16 AOM/DSS-induced carcinomas, nuclear or nuclear and cytoplasmatic beta-catenin expression was observed (**table 1**). The distribution of beta-catenin expression differed significantly between both groups (p = 0.020). In four out of seven non-malignant controls of AOM/DSS-colitis, membranous localization was observed, while the remaining three mucosa samples were characterized by membranous and cytoplasmatic expression, none showed nuclear expression. All four non-malignant controls of IL-10^−/−^ colitis presented membranous expression of beta-catenin.

In IL-10^−/−^ colitis as well as in IL-10^−/−^-induced tumors, very high levels of p53-protein expression were detected. In contrast, while p53 expression was still elevated in AOM/DSS-colitis, it was significantly below that observed in IL-10^−/−^ tumors (p = 0.010, **table 1**). In addition, premalignant mucosa of AOM/DSS-colitis revealed significantly lower p53-expression than AOM/DSS-induced CRCs (p = 0.015).

**Figure 3:**
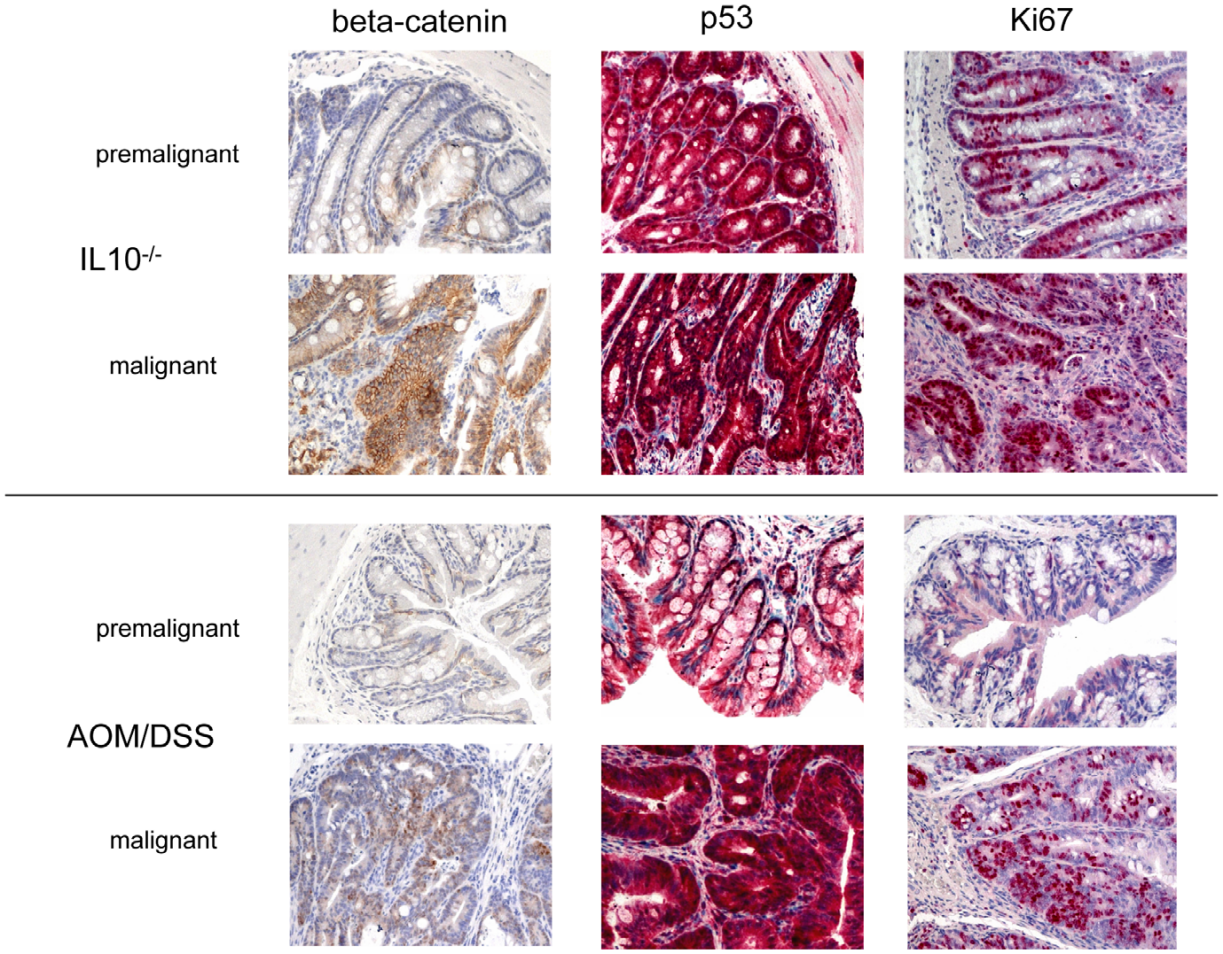
Immunohistochemistry for beta-catenin, p53 and Ki67 of murine premalignant tissue and CACs, details of 100x magnification.

Within the group of AOM/DSS-induced tumors, there was neither a significant correlation between the presence of aneuploidy and beta-catenin expression (p = 0.580) nor between aneuploidy and p53 expression (p = 0.730). The growth fraction of the tumor was generally higher in IL-10^−/−^-induced than in AOM/DSS-colitis-induced carcinomas (**table 1**). Thus, high expression of Ki67 was observed for premalignant mucosa of IL-10^−/−^ animals, while it was low in premalignant stages of AOM/DSS-induced colitis **(** **table 1** **,** **figure 3**).

## Discussion

Previously, a significantly higher frequency of aneuploidy reflecting chromosomal instability in human colitis-associated carcinomas as compared to the sporadic counterpart has been described [10]. To assess whether canonical mouse models of colitis-associated carcinogenesis exist that equally exhibit signs of CIN, we investigated ploidy patterns in murine colitis-associated neoplasms of two murine models of CAC.

In IL-10^−/−^ mice, neoplasms develop that resemble human adenocarcinomas, which has been described previously [26], [31], [32]. Severity of inflammation can be aggravated and malignant transformation accelerated by additional administration of celecoxib [25]. Carcinomas of the IL-10^−/−^ /celecoxib model did not exhibit signs of genomic instability in our study. Contrarily, tumors arising in the AOM/DSS-model presented with gross aneuploidy in the vast majority of cases. Although the number of specimens investigated differed between both groups with a considerably smaller number in the IL-10^−/−^-group, differences in the frequency of aneuploidy reached the level of significance. The presence of biological differences between CACs in both models is furthermore suggested by differential expression of beta-catenin.

In IL-10^−/−^ mice no extrinsic carcinogen is required to induce tumorigenesis. However, the complete absence of a critical cytokine for intestinal homeostasis represents an unphysiological state, even though altered IL-10 expression has previously been associated with human UC [33], [34]. Our data provide evidence that chronic inflammation due to IL-10 deficiency does not induce CIN, despite the presence of severe colonic inflammation. Moreover, further characterization of the tumors indicates that beta-catenin expression in premalignant stages and in carcinomas is confined to the cell membrane. This seems to stand in contrast to human CACs, in which APC mutations are thought to inhibit beta-catenin degradation and thereby promote nuclear translocation, although detailed data in humans is missing [18], whereas the high expression of phosphorylated p53 observed in these tumors is congruent with results in human UC-associated carcinomas [13].

In AOM/DSS colitis, an extrinsic carcinogen is needed to induce malignant transformation. Administration of DSS – and thereby initiation of chronic colitis – accelerates malignancy development, rendering the model particularly interesting for the study of colitis-associated carcinogenesis [22], [35]. Here, we could show that a high percentage of CACs that arise after AOM/DSS treatment exhibit CIN (81.25%). This finding is particularly remarkable since it has previously been demonstrated that CRCs induced by administration of AOM alone, which therefore arise devoid of chronic inflammation, are genetically stable [23]. Thus, it can be hypothesized that similar to human colitis, murine colonic inflammation induces or significantly contributes to CIN. AOM/DSS colitis might therefore provide a powerful model to study the development of inflammation-induced CIN *in vivo*. Furthermore, in AOM/DSS induced tumors, cytoplasmatic and/or nuclear translocation of beta-catenin was observed, indicating activation of the Wnt-signaling pathway [18], [36].

Expression of phosphorylated p53 was increased in AOM/DSS induced tumors, alongside with an increase in growth fraction as compared to premalignant stages. No association between the presence of aneuploidy and expression of p53 or beta-catenin could be shown, which might be due to insufficient numbers (n = 3 for the diploid group). Larger studies would be necessary to elucidate differential expression of tumor-associated proteins in relation to CIN in this murine model.

In neither of both models investigated in this study, macroscopic distant metastases could be observed. This is in line with previous findings [26], [37], but stands in contrast to CRCs in humans, in which liver metastases occur in up to 50% of patients during the course of the disease [38]. In this context, inevitable limitations to translate biological findings from mice into the human system have to be appreciated. For CIN in inflammation-associated carcinogenesis specifically, telomere shortening could be associated with increased genomic instability [39]. As differences exist between murine and human telomere biology with mice bearing longer telomeres and constitutive telomerase activity [40], it might be questionable to address telomere attrition in the AOM/DSS model. However, e. g. in murine epithelial cancers, telomere and telomerase dysfunction have been shown to be pivotal for tumor development, exemplifying a possible role for telomere biology in murine carcinogenesis [41]. Furthermore, other mechanisms such as chromatid cohesion defects have been associated with CIN in CRC, which involve proteins highly similar in man and mice and studied in both organisms [42], [43].

In summary, IL-10-deficient mice do not represent a suitable animal model for the study of the CIN pathway of carcinogenesis. In addition, tumor development in this model does not depend on beta-catenin translocation.

Contrarily, with presence of aneuploidy and beta-catenin activation, the model of AOM/DSS-colitis might provide a valuable tool to gain a more detailed insight into the molecular architecture of inflammation-associated carcinogenesis and mechanistically investigate colitis-associated carcinogenesis with special regard to CIN.
